# Supplementary figures and images for: Evaluating the effects of cardiometabolic exposures on circulating proteins which may contribute to severe SARS-CoV-2
Source: eBioMedicine. 2021 Feb 3;64:103228. doi: 10.1016/j.ebiom.2021.103228 (PMC7857697; doi:10.1016/j.ebiom.2021.103228)

**Supplementary Figures**

Supplementary Figure 1


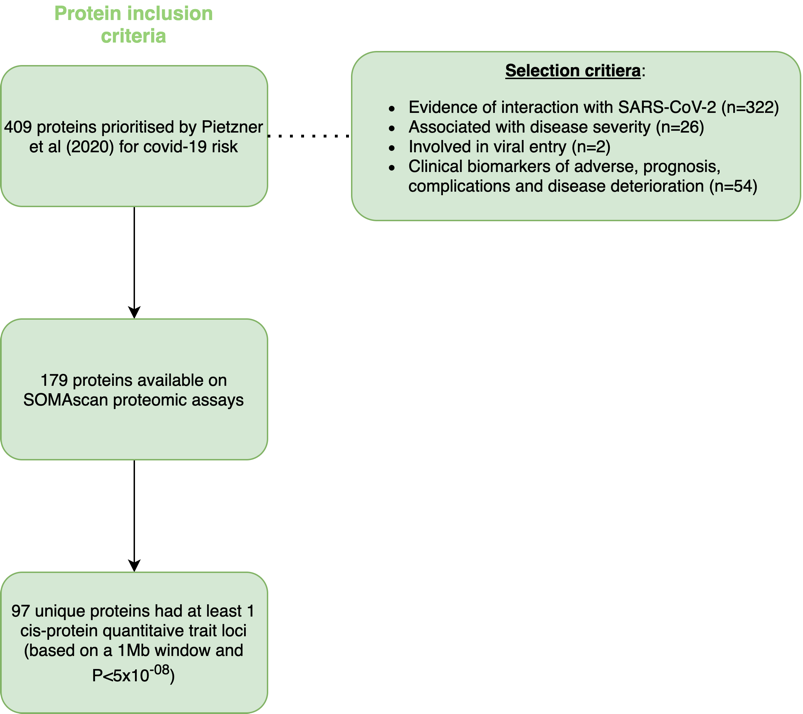


Supplementary Figure 2


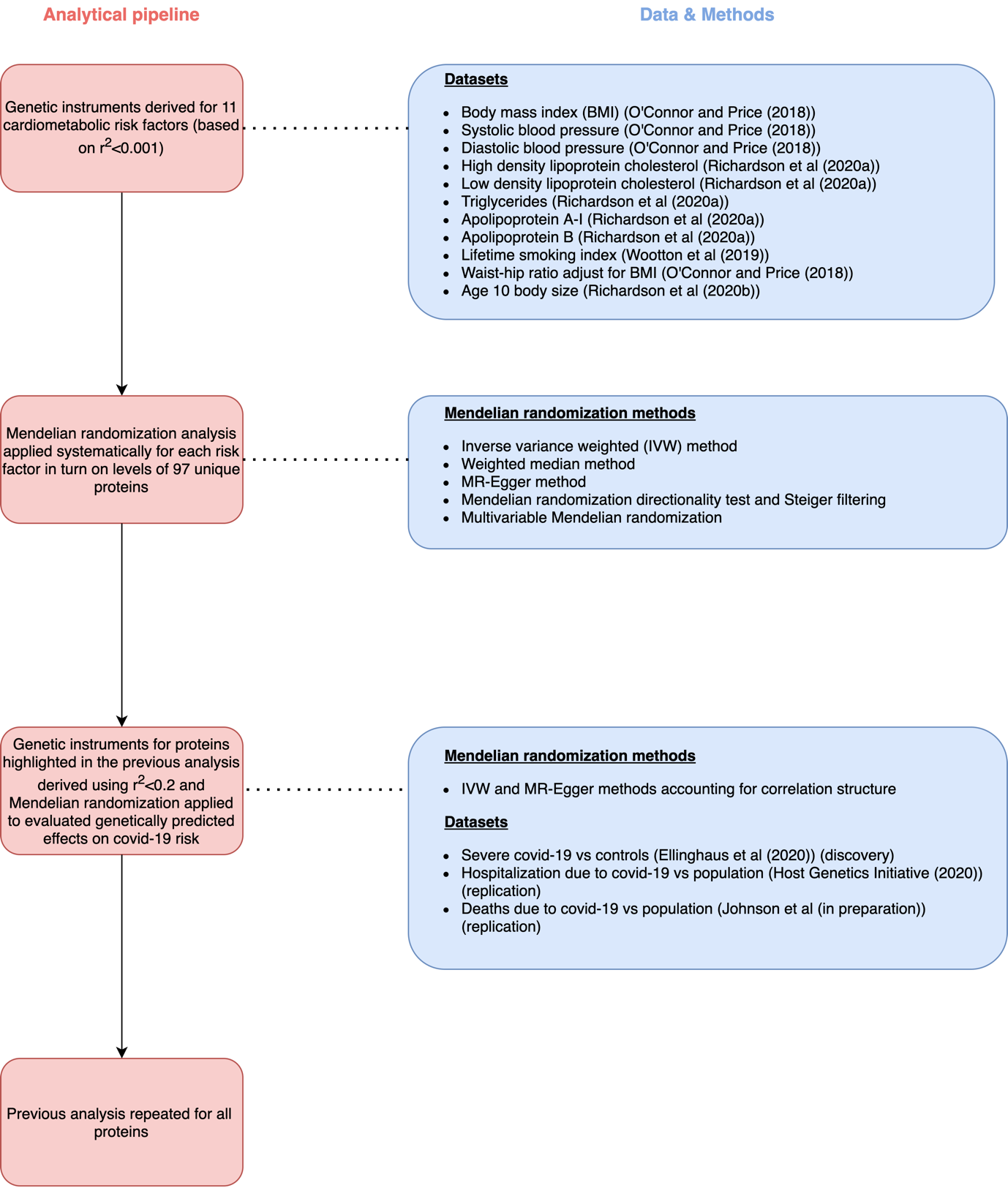

Supplement: Supplementary file 2 [file mmc2.docx]
